# A Rare Presentation of Renal Papillary Necrosis in a COVID-19-Positive Patient

**DOI:** 10.1155/2021/6611861

**Published:** 2021-01-11

**Authors:** Bela Tallai, Tawiz Gul Gulistan, Maged Nasser Aa B. Alrayashi, Salah Abdulhabeb Abdulwali Al Mughalles, Hatem Mostari Kamkoum, Mohamed Ali A. Ebrahim, Mohamed Abdelkarim Ali Abdelkarim, Morshed Ali Salah

**Affiliations:** ^1^Urology Division of Surgery Department, Hazm Mebaireek General Hospital, Hamad Medical Corporation, Industrial Area Street No. 33, PO Box 3050, Doha, Qatar; ^2^Clinical Imaging Department, Hazm Mebaireek General Hospital, Hamad Medical Corporation, Industrial Area Street No. 33, PO Box 3050, Doha, Qatar

## Abstract

In this case report we describe an unusual presentation of severe acute papillary necrosis in a COVID-19-positive patient. An emergency flexible ureteroscopy greatly helped in the establishment of the diagnosis. In the international literature, there is a paucity of intraoperative endoscopic images representing severe renal papillary necrosis. We present a case of severe acute renal papillary necrosis in a 49-year-old south-Asian, COVID-19-positive male patient who needed emergency urological intervention for macroscopic hematuria and urinary retention due to clot formation in the urinary bladder. The patient underwent emergency cystoscopy, clot evacuation, and by rigid and flexible ureteroscopy. The diagnosis was only confirmed in the postoperative period, retrospectively. Finally, the patient fully recovered due to the multidisciplinary management. Diagnosis of rare clinical entities can be sometimes challenging in the everyday routine practice. Having atypical clinical course, the surgeon should be prepared and sometimes must take responsible decisions promptly, even if needed intraoperatively, to manage unexpected findings in order to get the right diagnosis without compromising the patient's safety.

## 1. Introduction

Renal papillary necrosis is defined as an ischemic coagulative necrosis of the papillae in the kidneys. In most patients, diagnosis can be confirmed based on clinical symptoms and renal imaging [1]. A variety of etiological factors are known which can contribute to the process [2]. Clinical presentation can vary from an incidental finding in an asymptomatic patient to a severe, life-threatening condition. In the international literature, we did not find such intraoperative endoscopic images representing severe renal papillary necrosis we had obtained during the surgery.

In this case report, we describe a challenging clinical presentation of an acute papillary necrosis in a COVID-19-positive patient.

## 2. Case Presentation

A 49-year-old male patient with recent history of faint hematuria was admitted to a private outpatient clinic due to acute urinary retention. Apart from diabetes mellitus, his past medical and surgical histories were unremarkable. Foley catheter was smoothly inserted then frank bloody urine output could be observed. Therefore, he was referred to the governmental tertiary hospital as a urology emergency for further investigations and management. Besides faint hematuria and urinary retention, he did not have any other symptom. The patient was vitally stable and afebrile.

Physical examination of the genitourinary system was unremarkable. Among the initial laboratory investigations, mild anemia (hemoglobin 9.5 g/dL, hematocrit 29.5%) and azotemia (creatinine 153 *μ*mL/L) were noted. C-reactive protein (CRP) was also elevated (185 mg/L). Regarding imaging, kidney-ureter-bladder (KUB) ultrasound did not report any pathology at the level of the kidneys; only a large blood clot was described on the trigone of the bladder. Due to the elevated kidney function test, only a noncontrast computer tomography (CT) scan was carried out, which reported the same clot, suggesting some sort of bladder wall origin. The patient was admitted for emergency surgical procedure. The plan was to do cystoscopy, clot evacuation, and probably transurethral resection of the urinary bladder (TURB) or bladder biopsy with appropriate hemostasis. A COVID-19 polymerase chain reaction (PCR) swab test was ordered as well preoperatively showing positive result. Nevertheless, the patient did not have any symptoms suggestive of ongoing COVID-19 infection. Due to the positive swab test result, the patient was transferred to our designated COVID-19 hospital where only coronavirus-positive patients were hospitalized, and all emergency procedures were carried according to established local pathways and protocols. Upon the patient's arrival, Hgb dropped to 7.5 g/dL. In the operation theater, under spinal anesthesia, the clot was evacuated cystoscopically. Surprisingly, no source of bleeding could be observed during meticulous cystoscopy. At the final moments of cystoscopy, a thick bloody jet was expulsed from the left ureteric orifice which appeared repeatedly.

**Figure 1:**
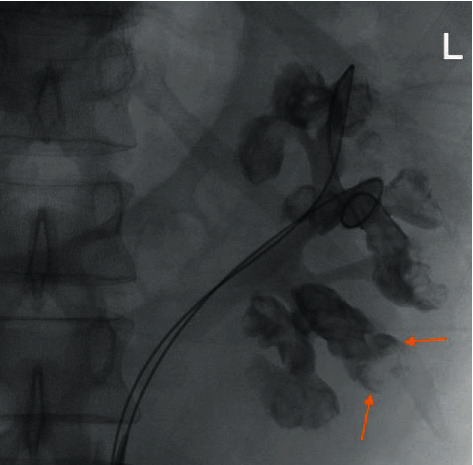
Left retrograde pyelography showing multiple filling defects in almost all caliceal ends. Arrows demonstrate the lobster claw sign as the characteristic radiology feature of renal papillary necrosis.

Having realized the unexpected intraoperative situation, left rigid and flexible ureteroscopy (FURS) were carried out. Rigid ureteroscopy was negative up to the pelvi-ureteric junction. Multiple filling defects in several caliceal endings were seen during retrograde pyelography (Figure 1).

**Figure 2:**
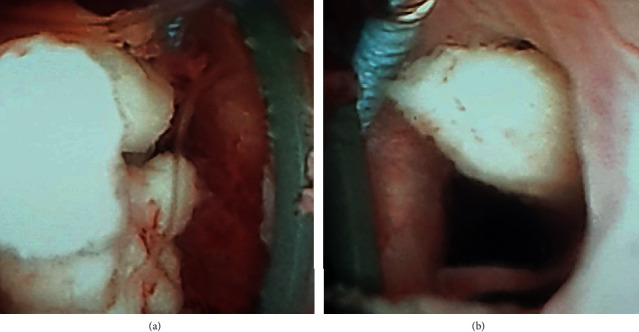
(a) Whitish, fluffy substance representing necrotic, sloughed papilla in inflammatory and hemorrhagic mucosal environment. (b) Sloughing necrotic papilla in the left-mid caliceal system.

During FURS, along with diffuse inflammatory and hemorrhagic mucosal changes in the calyces, whitish, fluffy substance was sitting in almost all renal calyceal ends (Figures 2(a) and 2(b)).

The renal pelvis was intact. Due to the bizarre intraoperative finding, multiple biopsies and urine cytology samples followed. By the end of the procedure, only a simple ureteral catheter was left behind for 24 hours to avoid further mucosal irritation and bleeding.

**Figure 3:**
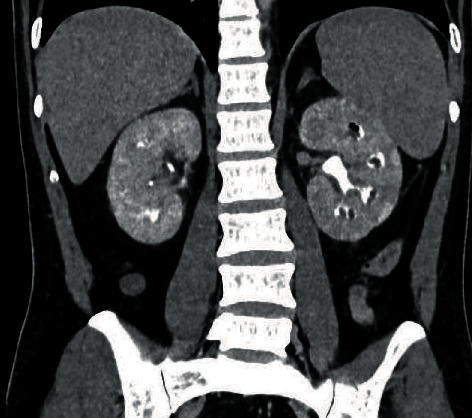
Renal papillary necrosis in the left kidney on delayed contrast CT scan on the 5^th^ postoperative day. Coronal reconstruction. Air in the upper caliceal system represents prior instrumentation.

The surgery went without any complication, and the postoperative period was uneventful as well. Finally, postoperative CT scan with contrast confirmed the diagnosis; however, it was already obvious for the radiologist when he received the images of intraoperative retrograde pyelography (Figure 3).

Later, the patient's condition continually improved, hematuria disappeared and the renal function became normal. The patient received 4 units of packed red blood cells during his hospital course. Both histology and cytology samples were negative for malignancy, showing necrotic debris with extensive neutrophilic infiltration, fibrin deposition, and acute inflammatory exudate. Investigations towards sickle cell anemia or tuberculosis were also negative. Nephrology was involved in the management in the postoperative period. However, though the patient's COVID-19 swab test was positive, he did not require any specific medication according to our infectious disease team. Finally, the patient was discharged into quarantine facility in fair general condition and with clear urine output.

During the multidisciplinary regular follow-up, the patient's urine has been clear, renal function has been maintained within the normal range, and blood sugar homeostasis has been controlled as well.

## 3. Discussion

Renal papillary necrosis is basically a rare medical disease. Several systemic factors can contribute in the development of the process, some of them might present simultaneously, synergizing each other [2]. The well-known underlying conditions are diabetes mellitus, analgesic abuse, sickle cell anemia, urinary tract obstruction, liver cirrhosis, and tuberculosis. The most common symptoms include flank and/or abdominal pain, various degrees of hematuria, dysuria, and chills and fever as signs of consecutive upper urinary tract infection [3]. In the presence of any clinical suspicion, usually, CT scan with contrast confirms the diagnosis and no further diagnostic or therapeutic interventions are required to be performed. In particular, during the COVID-19 pandemic, in selected patients, according to the individual institutional protocol, chest CT, in addition to abdominal scans, might have an important role in patients with the symptomatology of acute abdomen, even without respiratory symptoms [4].

The process of renal papillary necrosis can also show a chronic, slowly progressing course. Many of the patients can be treated conservatively without surgical intervention, and hematuria usually does not require blood transfusion. The single indication for urological intervention is acute ureteral obstruction by the necrotic tissue or blood clots or consecutive obstructive pyelonephritis [5]. Our patient deviated from most of the patients having renal papillary necrosis. He presented with isolated macroscopic hematuria, which progressed to clot urinary retention. No abdominal or flank pain, no fever or chills, and no signs of pyelonephritis could be observed either clinically or biochemically. Due to the continuous bleeding resulting in the significant drop in the hemoglobin level, the patient received blood transfusions and was taken to the operating theater on emergency basis. We expected that the patient had a sort of bladder pathology, because the clinical picture as well as the available preoperative imaging showed abnormality at the upper urinary tract beyond the bladder. Finally, FURS greatly helped us to confirm the diagnosis and completed the missing piece of the puzzle. However, the clinical manifestation, though it was somehow misleading, and laboratory and radiology findings retrospectively were relevant to the diagnosis. Among the well-known risk factors, type II diabetes mellitus was present.

Regarding the surgical process itself, wearing full personal protective equipment is a must in such emergency surgeries in a COVID-19-positive patient. However, due to these pieces of equipment, most of the surgeons have additional intraoperative burdens, negatively affecting their nontechnical skills, such as vision and communication, as well as impacting decision-making processes and also resulting in surgical discomfort and increased fatigue [6].

Another general debate or consideration is whether to perform a laparoscopic/endoscopic emergency surgery during the COVID-19 outbreak or to perform a traditional, appropriate open surgery, instead. Concerning the answer to this question, first of all, nonoperative management should represent the first line in a COVID-19 patient as far as possible. Open surgeries, if needed, might be recommended and should be performed safely in most patients especially in acute care surgery, avoiding the creation of pneumoperitoneum and other smoke-generating and time-consuming maneuvers, which might increase the risk of aerosolization during laparoscopy. Establishing local institutional treatment algorithms or protocols based on international guidelines is essential to manage these patients properly [7, 8]. In our case scenario, if we would have been faced with an uncontrollable bleeding with renal origin, we had also been challenged whether to treat it conservatively or with selective embolization or open surgery, as the last resort, depending mainly on the stability of the patient and the degree of bleeding. In a profuse, life-threating situation, a laparoscopic approach is definitely much less preferable for such patient. Fortunately, ureteroscopy in our patient was safe without increasing the chance of spreading the virus to the staff of the operating theater.

Our patient was COVID-19 positive; however, he was asymptomatic from this point of view and he did not receive any medication for that reason based on the medical COVID-19 protocol of our hospital. Probably, it will remain unknown how his viral infection impacted the basic necrotizing process in the kidneys. We considered his COVID-19 infection coincidental; nonetheless, its negative additional impact could not be excluded.

## 4. Conclusions

Our case demonstrated that sometimes, papillary necrosis can be a diagnosis of elimination or exclusion. This rare pathology should be suspected after excluding all other, much more common causes of hematuria. At the atypical clinical presentation, the insufficient preoperative imaging modalities can be misleading to and challenging for the urologist in obtaining the right diagnosis.

Based on our case report, we can also conclude that in such a severe pandemic, also with all its negative mental impact, it can be even more challenging for health care providers to stay alert and vigilant and at the same time make the right decision for cases that do not fit in the everyday routine practice.

## Data Availability

All the data related to the article can already be found in the manuscript.
